# Dual Antiplatelet Therapy in Patients With Minor Stroke Receiving Intravenous Thrombolysis

**DOI:** 10.3389/fneur.2022.819896

**Published:** 2022-02-04

**Authors:** Zhaohan Xu, Nihong Chen, Huiling Sun, Teng Jiang, Qiwen Deng, Junshan Zhou, Yingdong Zhang

**Affiliations:** ^1^Department of Neurology, Nanjing First Hospital, Nanjing Medical University, Nanjing, China; ^2^Department of Neurology, Nanjing Yuhua Hospital, Yuhua Branch of Nanjing First Hospital, Nanjing Medical University, Nanjing, China; ^3^General Clinical Research Centre, Nanjing First Hospital, Nanjing Medical University, Nanjing, China

**Keywords:** minor stroke, intravenous thrombolysis, dual antiplatelet, ischemic stroke, secondary prevention

## Abstract

**Introduction:**

Concern over the potential severe bleeding risk of dual antiplatelet therapy for patients with minor stroke after intravenous thrombolysis (IVT) leads to different antiplatelet strategies in the secondary prevention of stroke. Our aim was to investigate the effect of dual antiplatelet therapy on patients with minor ischemic stroke receiving IVT.

**Methods:**

From November 2016 to April 2021, a total of 855 consecutive patients who received IVT were observed. We collected and analyzed demographic characteristics, medical history, clinical information, and important time metrics of patients with minor ischemic stroke. Comparative and multivariate logistic regression analyses were used to explore the clinical significance of single or dual antiplatelet therapy after IVT. Propensity score matching analyses (1:1 matching including baseline characteristics of patients) were also performed.

**Results:**

A total of 245 patients were enrolled in the study (118 patients in the single antiplatelet therapy group and 127 patients in the dual antiplatelet group). No significant difference was found in baseline characteristics except stroke etiology (*p* < 0.001) for patients with minor stroke. The dual antiplatelet group showed a higher proportion of 90-day modified Rankin Scale (mRS) (0–1) than the single antiplatelet group (*p* = 0.030). Furthermore, patients receiving dual antiplatelet therapy had excellent outcomes (90-day mRS 0–1) after adjustment (odds ratio [*OR*] 2.76, 95% *CI* 1.27–6.01, *p* = 0.010). Other secondary outcomes (recurrent stroke within 90 days, symptomatic intracerebral hemorrhage, and early neurological deterioration) were not significantly different between the two groups. These findings were generally consistent in propensity score analyses.

**Conclusions:**

Dual antiplatelet therapy may be a potential therapeutic approach in patients with minor stroke receiving IVT. Further randomized controlled trials are required to confirm this finding.

## Introduction

The high incidence, disability rate, and mortality of ischemic stroke place a heavy burden on families and societies around the world (1–3). Mounting evidence has proven that intravenous thrombolysis (IVT) (recombinant tissue plasminogen activator-PA, rt-PA) and endovascular thrombectomy are effective methods to gain vascular recanalisation and restore blood flow (4–8). Furthermore, administration of antiplatelets is recommended for eligible patients with AIS within 24–48 h after onset (3). These methods are dedicated for improving the prognosis of patients with acute ischemic stroke (AIS) and reducing the possibility of stroke recurrence.

Minor stroke is commonly considered a minor neurological deficit with a low National Institutes of Health Stroke Scale (NIHSS) score. Although definitions of low NIHSS scores vary from studies with a range of 0~7 scores, a low NIHSS score ≤ 3 is commonly considered a minor stroke in clinical trials and studies (9). It has been reported that these patients (NIHSS ≤ 3) make up more than half of all ischemic stroke cases (10). For patients with mild but disabling stroke symptoms, IV alteplase is also considered reasonable for use by the guidelines, as there is proven clinical benefit for eligible patients with minor stroke (3, 11, 12). Although a relatively low NIHSS score may be considered disabling, trials have shown a benefit for IV alteplase, and studies have indicated that clinical functional outcomes and risks were the same in mild stroke treated within a time window with or without the categorization as disabling neurological deficits (13–15). The CHANCE trial fundamentally indicated the benefit of clopidogrel and aspirin dual antiplatelet treatment within 24 h from symptoms onset for patients with minor stroke (NIHSS ≤ 3) without thrombolysis by reducing the risk of subsequent stroke (16). The POINT trial found that the short-term (21-day) treatment with clopidogrel and aspirin reduced the risk of major ischemic events without increasing the risk of major hemorrhage at 90 days (17). Both the CHANCE trial and the POINT trial focused on patients with minor stroke without thrombolysis. Guidelines have specifically recommended the administration of dual antiplatelet therapy for 10–21 days for patients with minor stroke (NIHSS ≤ 3) (18), based on the findings from clinical trials. However, there are quite a number of minor stroke patients who actually receive thrombolysis treatment due to the reasonable recommendation of the guidelines. Nevertheless, both rt-PA thrombolytic therapy and antiplatelet treatment have potential bleeding risks and other complications (19–22), which are worrying in real-world practice, leading to different strategies of antiplatelet treatment for patients with minor stroke receiving IVT. A previous study reported the safety and efficiency of 21-day dual antiplatelet therapy in patients who received IVT compared with single antiplatelet therapy (23). However, no guidelines or large randomized clinical trials have yet identified an antiplatelet strategy under this scenario. The influence of single or dual antiplatelet therapy on patients with minor stroke receiving IVT remains uncertain.

In the present article, we utilized existing cases and conducted a retrospective study to analyse the effects of single or dual antiplatelet therapy on clinical outcomes in minor stroke patients receiving IVT.

## Methods

### Study Subjects and Antiplatelet Therapy Strategies

From November 2016 to April 2021, we conducted this retrospective study in the National Advanced Stroke Center of Nanjing First Hospital, Nanjing Medical University, a national advanced stroke center affiliated with the Stroke Prevention Project of the National Health Commission. We continuously observed 859 patients with acute stroke receiving IVT within the 4.5 h stroke onset to treatment time window. When considering the guidelines, clinical reports, and actual conditions, we explained thrombolysis for patients with typical mild stroke with functional symptoms when current guidelines, principles, benefits, and potential risks were fully explained to patients and their families. An informed consent was signed before thrombolysis treatment. In this study, patients who were fully aware of the current reasonable indications and who received IVT with a low NIHSS score ( ≤ 3) at admission were recruited and prescribed antiplatelet therapy for at least 21 days. In our stroke center, we have mainly developed two strategies of antiplatelet therapy for patients with minor stroke who received thrombolysis, referring to the guidelines for the early management of patients with AIS and real-world clinical concerns. One is using a single antiplatelet drug (aspirin 100 mg/day or clopidogrel 75 mg/day), while the other is dual antiplatelet (aspirin 100 mg/day and clopidogrel 75 mg/day) therapy. In this study, we enrolled patients with NIHSS scores ≤ 3 at admission in accordance with the CHANCE (16) and POINT trials (17). Patients with atrial fibrillation during follow-up were accessed and excluded by conducting serial Holter ECGs. Both groups receive antiplatelet therapy 24 h after thrombolysis without intracranial bleeding. The detailed inclusion criteria were as follows: (1) AIS symptoms occurring within 4.5 h and receiving IVT [rt-PA 0.9 mg/kg]; (2) NIHSS score ≤ 3 at admission; (3) age over 18 years; and (4) single or dual antiplatelet therapy for 21 days. The exclusion criteria were as follows: (1) NIHSS score > 3 at admission; (2) history of atrial fibrillation or diagnosis of cardioembolic stroke; (3) CT indicated intracranial bleeding after thrombolysis; (4) absence of complete follow-up; and (5) absence of informed consent to join the study. The primary objective was to assess the effects of the two treatment groups on the incidence of recurrent stroke and symptomatic intracerebral hemorrhage in the first 90 days after acute minor stroke. Moreover, we observed whether there were differences in patients' 90-day outcomes and early neurological deterioration under these two strategies.

### Clinical Assessments

Baseline demographic characteristics and important time metrics of patients were collected by our fast-response stroke care unit, such as professional neurologists and stroke nurses. The condition of each patient was assessed strictly and documented at admission and at 24 h, 3 and 7 days with the NIHSS. Stroke etiology was assessed by the Trial of ORG 10,172 in Acute Stroke Treatment (TOAST) classification (24). A follow-up 90 days after discharge was arranged face-to-face or over the phone using the modified Rankin Scale (mRS) (25).

### Statistical Analysis

Categorical variables are expressed as frequencies and percentages. Normally distributed variables are expressed as the mean ± SD, and abnormally distributed continuous variables are expressed as medians (interquartile range [IQR]). Comparisons were made between two groups using the 2-tailed *t*-tests and Mann–Whitney *U*-tests. Variables from significant factors of the univariable logistic regression analysis (*p* < 0.01) were further analyzed by multivariate logistic regression to obtain independent variables. The significance threshold was set at <0.05 (*p* < 0.05). A propensity score-matching (PSM) analysis was also used to obtain the matched pairs of samples from patients with single or dual antiplatelet treatment. In the PSM algorithm, the corresponding propensity score of the grouping variable (single or dual antiplatelet treatment) was calculated for each patient by using a 1:1 nearest-neighbor matching algorithm, with a caliper width of 0.2 of the propensity score and with age, sex, medical histories (previous histories of ischemic stroke, diabetes mellitus, and hypertension), mRS score before stroke, median glucose level at admission, Oxfordshire Community Stroke Project (OCSP) classification, stroke etiology, important time metrics at admission, and NIHSS score before IVT as covariates.

### Ethics Statements

This study policy was explained in detail, and informed consent was obtained from each study patient or their family members. This whole study was reviewed and approved by the ethics committee of Nanjing First Hospital, Nanjing Medical University and conducted in full accordance with the World Medical Association Declaration of Helsinki.

## Results

**Figure 1:**
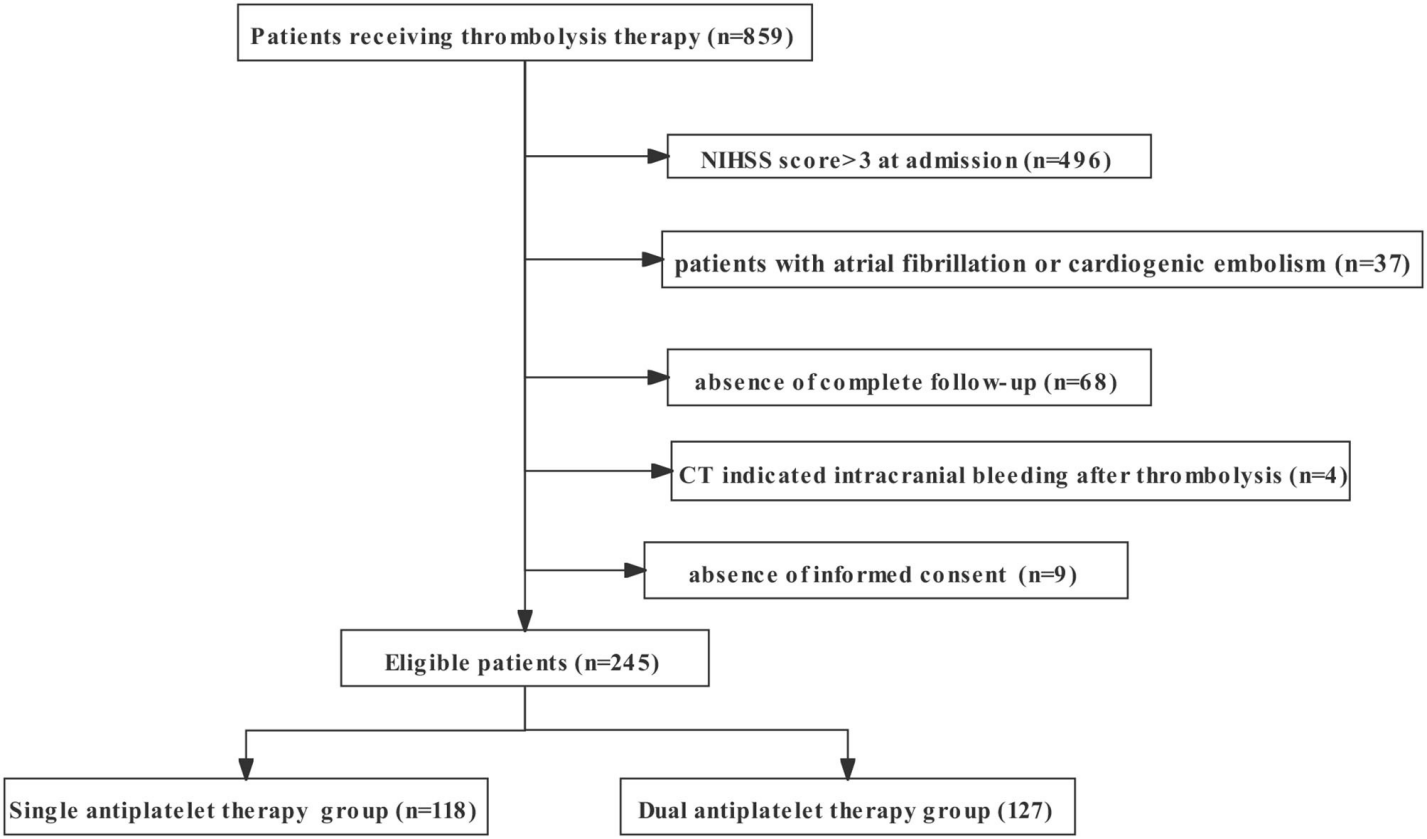
The flowchart of recruitment information on patients enrolled in the study. NIHSS, National Institutes of Health Stroke Scale.

Of the 859 screened patients receiving thrombolysis treatment in our center from November 2016 to April 2021, we excluded 496 patients with moderate or severe stroke (NIHSS score >3), 37 patients with a history of atrial fibrillation or diagnosis of cardioembolic stroke, 4 patients with intracranial bleeding before antiplatelet treatment, 68 patients without complete follow-up, and nine patients without consent to join the study. A total of 245 patients met the inclusion criteria (118 patients were enrolled in the single antiplatelet therapy group, and 127 patients were enrolled in the dual antiplatelet therapy group). Detailed recruitment information is shown in Figure 1. The baseline demographic characteristics of the eligible patients are summarized in Table 1.

**Table 1:** Baseline demographic characteristics.

	**All (245)**	**Single antiplatelet group (118)**	**Dual antiplatelet group (127)**	***p***
Mean age (SD)-yr.	66 (11.9)	67 (12.5)	66 (11.4)	0.152
Male sex-no. (%)	183 (74.69)	86 (72.88)	97 (76.38)	0.529
Medical history-no. (%)				
Previous ischemic stroke	29 (11.84)	13 (11.02)	16 (12.60)	0.702
History of diabetes mellitus	71 (28.98)	36 (30.51)	35 (27.56)	0.611
History of hypertension	182 (74.29)	84 (71.19)	98 (77.17)	0.285
Median mRS score before stroke onset (IQR)-point	0 (0–0)	0 (0–0)	0 (0–0)	0.437
mRS score point 0~1 before stroke-no. (%)	234 (95.51)	111 (94.07)	123 (96.85)	0.293
Median glucose level at admission (IQR)-mmol/L	5.13 (4.52–6.20)	5.05 (4.56–6.19)	5.16 (4.49–6.20)	0.909
*OCSP classification*				0.629
PACI	209 (85.31)	102 (86.44)	107 (84.25)	
POCI	36 (14.69)	16 (13.56)	20 (15.75)	
*Cause of stroke - no. (%)*				<0.001
Large-artery atherosclerosis	76 (31.02)	21 (17.80)	55 (43.31)	
Small-vessel occlusion	159 (64.90)	92 (77.97)	67 (52.76)	
Stroke of undetermined etiology	10 (4.08)	5 (4.24)	5 (3.94)	
*Median duration (IQR)-min*				
From stroke onset to hospital admission	115 (60–160)	118 (60–160)	110 (70–160)	0.792
From hospital admission to IVT	27 (20–40)	27 (20–40)	27 (20–40)	0.496
From stroke onset to IVT	145 (105–198)	150 (100–196)	145 (108–200)	0.793
NIHSS score				
Median NIHSS score (IQR) before IVT	2 (2–3)	2 (2–3)	2 (1–3)	0.260
Median NIHSS score (IQR) at 1h after IVT	2 (1–3)	2 (1–3)	2 (1–2)	0.068
Median NIHSS score (IQR) at 24h after IVT	1 (1–2)	1 (1–2)	1 (0–2)	0.070
Median NIHSS score (IQR) at 3d after IVT	1 (0–2)	1 (0–2)	1 (0–2)	0.421
Median NIHSS score (IQR) at 7d after IVT	1 (0–1)	1 (0–1)	1 (0–1)	0.932

*IVT, intravenous thrombolysis; NIHSS, National Institute of Health Stroke Scale; OCSP Oxfordshire Community Stroke Project; PACI, partial anterior circulation infarcts; POCI, posterior circulation infarcts based on the Trial of ORG 10,172 in Acute Stroke Treatment (TOAST) classification*.

There was no significant difference between the single and dual antiplatelet therapy groups in the patients' gender, age, relevant medical history, median glucose level at admission, initial mRS, NIHSS score at admission, or important time metrics in the early management of ischemic stroke. However, the two groups showed different proportion ratios of the cause of stroke (*p* < 0.001). There was mainly no difference in the median NIHSS score in the early stage of hospitalization. The primary outcome showed that no difference was found in the recurrence of stroke within 90 days, symptomatic intracerebral hemorrhage, or early neurological deterioration between the two groups.

The secondary outcomes, however, indicated a better overall 90-day mRS score in the dual antiplatelet group (*p* = 0.012). Clinical outcomes of the 90-mRS score and the range of 90-mRS score are shown in Table 2. In addition, there were more patients with 90-day mRS (0–1) in the dual antiplatelet group (*p* = 0.030). Detailed information of multivariate logistic regression analysis is shown in Table 3. After adjustment for age, sex, and cause of stroke, patients receiving dual antiplatelet therapy had better overall 90-day mRS scores (odds ratio [*OR*] 0.71, 95% CI 0.55–0.92) and excellent recovery from stroke (90-day mRS 0-1) (*OR* 2.76, 95% *CI* 1.27–6.01, *p* = 0.010).

**Table 2:** Outcomes.

	**All (245)**	**Single antiplatelet group (118)**	**Dual antiplatelet group (127)**	***p***
**Primary outcome**				
Recurrence of stroke within 90 days	8 (3.27)	4 (3.39)	4 (3.15)	0.916
Symptomatic intracerebral hemorrhage-no. (%)	3 (1.22)	2 (1.69)	1 (0.79)	0.610
**Secondary outcomes**				
90-day mRS score	0 (0–1)	0 (0–1)	0 (0–1)	0.012
90-day mRS score 0–1	206 (84.08)	93 (78.81)	113 (88.98)	0.030
90-day mRS score 0–2	222 (90.61)	103 (87.29)	119 (93.70)	0.086
90-day mRS score 0–3	233 (95.10)	111 (94.07)	122 (96.06)	0.470
Early neurological deterioration no. (%)				
3d after IVT	14 (5.71)	8 (6.78)	6 (4.72)	0.489
7d after IVT	13 (5.31)	8 (6.78)	5 (3.94)	0.321

*mRS, modified Rankin Scale*.

**Table 3:** Multivariate logistic regression analysis of clinical outcomes after single or dual antiplatelet treatment.

**Outcome**	**Unadjusted effect (95% CI)**	***P***	**Adjusted effect (95% CI)**	***p***
Mean age (SD)-yr.	0.99 (0.97–1.02)	0.578	1.00 (0.97–1.02)	0.685
Male sex-no. (%)	1.20 (0.68–2.14)	0.530	1.30 (0.70–2.42)	0.403
*Cause of stroke - no. (%)*				
Large-artery atherosclerosis	1			
Small-vessel occlusion	0.28 (0.15–0.50)	<0.001	0.23 (0.12–0.43)	<0.001
undetermined	0.38 (0.10–1.45)	0.158	0.28 (0.07–1.13)	0.075
mRS at 90 days*	0.79 (0.62–0.99)	0.042	0.71 (0.55–0.92)	0.009
90-day mRS score 0–1*	2.17 (1.07–4.41)	0.032	2.76 (1.27–6.01)	0.010
90-day mRS score 0–2*	2.17 (0.88–5.32)	0.091		
90-day mRS score 0–3	1.54 (0.47–4.99)	0.473		
Recurrence of stroke within 90 days	0.93 (0.23–3.79)	0.916		
Early neurological deterioration no. (%)				
3d after IVT	0.68 (0.23–2.03)	0.491		
7d after IVT	0.56 (0.18–1.77)	0.327		
Symptomatic intracerebral hemorrhage-no. (%)	0.46 (0.04–5.14)	0.529		

*IVT, intravenous thrombolysis; NIHSS, National Institute of Health Stroke Scale, mRS, modified Rankin Scale based on the TOAST classification*.

*Adjusted with age, sex, and cause of stroke.

After the PSM analysis, all the baseline characteristics were well balanced, and 87 patients in each group (single and dual antiplatelet groups) were matched and compared for outcomes. Baseline characteristics and outcomes in the matched analyses are presented as supplemental materials in Table 4. The results were generally consistent with the primary analysis; no difference was found in the recurrence of stroke, symptomatic intracerebral hemorrhage, or early neurological deterioration, and the dual antiplatelet group showed a better overall 90-day mRS score (*p* < 0.001) and a higher proportion of 90-day mRS score (0–1) (*p* = 0.001). However, there were more patients in the specific 90-day mRS score range of 0–2 (*p* = 0.005).

**Table 4:** Baseline demographic characteristics and outcomes in the matched analyses.

	**All (174)**	**Single antiplatelet group (87)**	**Dual antiplatelet group (87)**	***p***
Median age (IQR)-yr.	66 (58–74)	65 (57–77)	66 (60–72)	0.865
Male sex-no. (%)	136 (78.16)	66 (75.86)	70 (80.46)	0.463
Medical history-no. (%)				
Previous ischemic stroke	22 (12.64)	11 (12.64)	11 (12.64)	1
History of diabetes mellitus	53 (30.46)	26 (29.89)	27 (31.03)	0.869
History of hypertension	130 (74.71)	64 (73.56)	66 (75.86)	0.727
Median mRS score before stroke onset (IQR)-point	0 (0–0)	0 (0–0)	0 (0–0)	0.945
mRS score point 0~1 before stroke-no. (%)				
Median glucose level at admission (IQR)-mmol/L	5.17 (4.51–6.13)	5.09 (4.46–6.28)	5.19 (4.55–5.95)	0.795
*Location of obstruction*				1
Anterior circulation	148 (85.06)	74 (85.06)	74 (85.06)	
Posterior circulation	26 (14.94)	13 (14.94)	13 (14.94)	
*Cause of stroke - no. (%)*				0.927
Large-artery atherosclerosis	42 (24.14)	21 (24.14)	21 (24.14)	
Small-vessel occlusion	125 (71.84)	63 (72.41)	62 (71.26)	
Stroke of undetermined etiology	7 (4.02)	3 (3.45)	4 (4.60)	
*Median duration (IQR)-min*				
From stroke onset to hospital admission	110 (60–160)	105 (60–150)	120 (65–180)	0.245
From hospital admission to IVT	27 (20–40)	28 (20–39)	27 (20–42)	0.604
From stroke onset to IVT	145 (100–196)	140 (92–190)	145 (107–202)	0.282
NIHSS score				
Median NIHSS score (IQR) before IVT	2 (1–3)	2 (1–3)	2 (1–3)	0.960
Median NIHSS score (IQR) at 1h after IVT	2 (1–3)	2 (1–3)	2 (1–2)	0.441
Median NIHSS score (IQR) at 24h after IVT	1 (1–2)	1 (1–2)	1 (1–2)	0.193
Median NIHSS score (IQR) at 3d after IVT	1 (0–2)	1 (0–2)	1 (0–2)	0.794
Median NIHSS score (IQR) at 7d after IVT	1 (0–1)	1 (0–1)	1 (0–1)	0.772
Recurrence of stroke within 90 days	6 (3.45)	4 (4.60)	2 (2.30)	0.678
Symptomatic intracerebral hemorrhage-no. (%)	3 (1.72)	2 (2.30)	1 (1.15)	0.560
90-day mRS score	0 (0–1)	0 (0–1)	0 (0–0)	<0.001
90-day mRS score 0–1	152 (87.36)	69 (79.31)	83 (95.40)	0.001
90-day mRS score 0–2	160 (91.95)	75 (86.21)	85 (97.70)	0.005
90-day mRS score 0–3	167 (95.98)	82 (94.25)	85 (97.70)	0.247
*Early neurological deterioration no. (%)*				
3d after IVT	10 (5.75)	7 (8.05)	3 (3.45)	0.193
7d after IVT	9 (5.17)	7 (8.05)	2 (2.30)	0.087

*IVT, intravenous thrombolysis; NIHSS, National Institute of Health Stroke Scale; OCSP Oxfordshire Community Stroke Project; PACI, partial anterior circulation infarcts; POCI, posterior circulation infarcts; mRS, modified Rankin Scale based on the TOAST classification*.

## Discussion

In this study, for patients with minor stroke receiving IVT, those who received 21-day dual antiplatelet (aspirin and clopidogrel) treatment showed a better 90-day mRS score than those who received single antiplatelet therapy. Meanwhile, no significant difference was found in the hemorrhage risk, early neurological deterioration, or stroke recurrence possibility.

There have been various definitions of minor stroke reported in different studies. However, minor stroke is mostly considered to demonstrate a low NIHSS score ( ≤ 3) (9, 26). The updated guidelines released in 2019 recommend that alteplase may be reasonable for patients with mild disabling stroke symptoms. In real-world clinical practice, we have gradually recognized that the NIHSS score is not strictly related to clinical outcomes (3, 26, 27). Symptoms of posterior circulation and right hemisphere are usually hard to capture by NIHSS. There is also a fairly large number of patients with low NIHSS scores at admission but low intracranial perfusion or brain injury, which is often detected by multimodal imaging (28). Thus, studies have confirmed that aggressive early management with thrombolysis treatment is beneficial for restoring the intracranial perfusion and improving the prognosis of minor stroke patients without increasing the risks of bleeding or other side effects (12, 29, 30).

For minor stroke patients without thrombolysis treatment, the CHANCE and the POINT trials demonstrated that treatment for 21 days with dual antiplatelet therapy (aspirin and clopidogrel) that is initiated within 24 h from symptom onset can be beneficial for the prevention of symptom recurrence for up to 90 days. Moreover, there are quite a number of patients with minor stroke who actually receive thrombolysis treatment due to the reasonable recommendations of the guidelines. In addition, both rt-PA thrombolytic therapy and antiplatelet drugs have potential bleeding risks, thus leading to concerns of side effects, especially for aspirin and clopidogrel dual antiplatelet therapy. To date, neither the guidelines nor randomized clinical trials have specifically illustrated the safety and necessity of dual antiplatelet therapy for patients with minor stroke patients who receive thrombolysis.

As a result, many neurologists tend to conservatively use single antiplatelet therapy (aspirin or clopidogrel) for patients with minor stroke who received thrombolysis (31). We noticed an article published by Dr. Zhang (23) in which the authors verified the efficacy and safety of dual antiplatelet therapy after IVT to some extent. They enrolled patients with acute minor ischemic stroke with a NIHSS score ≤ 5. Concerns on the possible bias were aroused when we noticed that some patients diagnosed with atrial fibrillation or cardiogenic embolism were included in this study. However, anticoagulants are generally recommended for those patients rather than antiplatelet agents to reduce stroke risk (32–34). In our study, we recruited patients with a low NIHSS score of ≤ 3 and excluded patients with a history of atrial fibrillation or diagnosis of cardiogenic embolism in accordance with the CHANCE trial and the guidelines of secondary stroke prevention for patients with minor stroke (18, 35). In our stroke center, antiplatelet therapy was followed for these patients with minor stroke 24 h after IVT with the exclusion of intracranial hemorrhage. In this retrospective study, we used existing data and information by carefully recording antiplatelet strategies of patients with minor stroke, and we also analyzed their functional recovery, 90-day mRS score, stroke recurrence, and hemorrhage risk to discuss the differences between single and dual antiplatelet therapies, which we hope may provide some reference value for the future clinical work. However, we admit that large randomized clinical trials are needed in the future to resolve any gaps in our study.

In addition, a significant difference was observed in the TOAST classification between the two groups: the dual antiplatelet therapy group had a higher rate of large artery atherosclerosis etiology in this study. Therefore, higher rates of recurrence could have been expected in the dual antiplatelet group (36, 37). There was no difference regarding the stroke recurrence rate between the two groups in our study, which suggests that dual antiplatelet therapy after thrombolysis therapy may be beneficial to the patients with minor stroke with large artery atherosclerosis. We assumed that the dual antiplatelet therapy may be more effective to reduce the stroke recurrence by inhibiting platelet adhesiveness and reducing the atherogenesis in intracranial vessels (38, 39). Although there were more patients with large artery atherosclerosis in the dual antiplatelet group, the dual antiplatelet group showed a better overall 90-day mRS score after adjustment in the multivariate logistic regression analysis. We hypothesized that the relatively aggressive antiplatelet therapy may helpfully delay the deterioration of blood vessels and the progression of stroke, which needs further studies to confirm.

In addition, we conducted a propensity score-matching analysis to reduce potential confounders. We assessed the balance of covariates that was achieved from matching by evaluating standardized differences between the single and dual antiplatelet groups. Eighty-seven pairs of patients were included in the matched cohorts. The results were generally consistent with the primary analysis and are shown in Table 4 as supplemental materials.

Several limitations should be acknowledged. First, this is a retrospective study from a single center. Although many patients were observed and analyzed in this study, there was a possibility of bias due to the selection and assessment of patients. Second, quite a number of patients in the 90-day follow-up could not clearly recall the incidences of gastrointestinal bleeding or skin or mucous membrane bleeding, which may have caused a memory bias. Additionally, we found that many patients were prescribed acid inhibitor, such as rabeprazole, to prevent gastrointestinal mucosa injury in advance. Thus, we did not include complications of extracranial hemorrhage as being observed, which requires more research in the future. Third, there are no relevant reports on whether a loading dose of antiplatelet therapy should be used 24 h after thrombolytic therapy, which needs further discussion and confirmation.

## Conclusion

Dual antiplatelet therapy may be a potential therapeutic strategy for patients with minor stroke who receive IVT. Further larger studies are needed to confirm this finding.

## Data Availability Statement

The original contributions presented in the study are included in the article/supplementary material, further inquiries can be directed to the corresponding authors.

## Ethics Statement

The studies involving human participants were reviewed and approved by the Ethics Committee of Nanjing First Hospital, Nanjing Medical University. The patients/participants provided their written informed consent to participate in this study.

## Author Contributions

YZ, JZ, and QD contributed to the study conception and design. ZX, NC, and HS performed the material preparation, data collection, and analysis. ZX and QD wrote the first draft of the manuscript. TJ reviewed and modified the manuscript and all authors commented on previous versions of the manuscript. All authors have read and approved the final manuscript.

## Funding

This work was supported by the National Natural Science Foundation of China (No. 81901215); the Stroke Prevention Project of the National Health Commission of the People's Republic of China (GN-2020R0013); the Medical Scientific Research Project of Jiangsu Commission of Health (ZDA2020019); the Health China BuChang ZhiYuan Public welfare projects for Heart and brain health (No. HIGHER202102); and the National Science and Technology Innovation 2030 – Major Program of Brain Science and Brain-Inspired Intelligence Research (2021ZD0201807).

## Conflict of Interest

The authors declare that the research was conducted in the absence of any commercial or financial relationships that could be construed as a potential conflict of interest.

## Publisher's Note

All claims expressed in this article are solely those of the authors and do not necessarily represent those of their affiliated organizations, or those of the publisher, the editors and the reviewers. Any product that may be evaluated in this article, or claim that may be made by its manufacturer, is not guaranteed or endorsed by the publisher.
